# Supplementary material for: X-ray study of ferroic octupole order producing anomalous Hall effect
Source: Nat Commun. 2021 Sep 22;12:5582. doi: 10.1038/s41467-021-25834-7 (PMC8458343; doi:10.1038/s41467-021-25834-7)
Supplement: Supplementary file 1 — Supplementary Information [file 41467_2021_25834_MOESM1_ESM.pdf]

Supplementary Information for

"X-ray study of ferroic octupole order producing anomalous Hall effect"

Motoi Kimata[1*], Norimasa Sasabe[2], Kensuke Kurita[3], Yuichi Yamasaki[4,5,6,7], Chihiro Tabata[8], Yuichi Yokoyama[2], Yoshinori Kotani[2], Muhammad Ikhlas[9], Takahiro Tomita[9], Kenta Amemiya[7], Hiroyuki Nojiri[1], Satoru Nakatsuji[9,10,11,12], Takashi Koretsune[3], Hironori Nakao[7], Taka-hisa Arima[5,13], & Tetsuya Nakamura[1,2,14]

[1]*Institute for Materials Research, Tohoku University, Sendai, Miyagi 980-8577, Japan*

[2]*Japan Synchrotron Radiation Research Institute (JASRI), 1-1-1 Kouto, Sayo, Hyogo 679-5198, Japan*

[3]*Depertment of Physics, Tohoku University, Sendai, Miyagi, Japan*

[4]*Research and Services Division of Materials Data and Integrated System (MaDIS), National Institute for Materials Science (NIMS), Tsukuba, Ibaraki, Japan*

[5]*Center for Emergent Matter Science (CEMS), RIKEN, Wako 351-0198, Japan*

[6]*PRESTO, Japan Science and Technology Agency (JST)*

[7]*Institute of Materials Structure Science, High Energy Accelerator Research Organization, Tsukuba, Ibaraki 305-0801, Japan*

[8]*Institute for Integrated Radiation and Nuclear Science, Kyoto University, Kumatori, Osaka 590-0494, Japan*

[9]*Institute for Solid State Physics, University of Tokyo, Kashiwa, Chiba, Japan*

[10]*Depertment of Physics, University of Tokyo, Hongo, Tokyo, Japan*

[11]*The Institute for Quantum Matter, Johns Hopkins University, Baltimore, MD 21218, USA*

[12]*Trans-scale Quantum Science Institute, University of Tokyo, Hongo, Tokyo, Japan*

[13]*Department of Advanced Materials Science, University of Tokyo, Kashiwa 277-8561, Japan*

[14]*International Center for Synchrotron Radiation Innovation Smart, Tohoku University, Sendai, Miyagi 980-8577, Japan*

* motoi.kimata.b4@tohoku.ac.jp

1. Magnetization data of Mn_3Sn single crystal

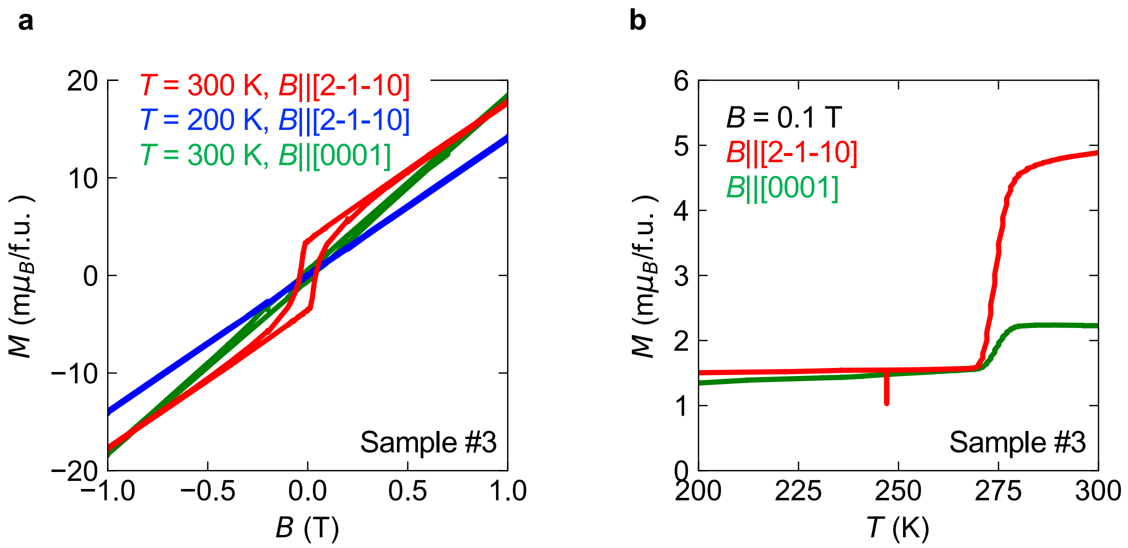

Supplementary Figure S1| Field strangle and temperature dependences of magnetization. a, Field-dependent magnetization curve for different field directions and temperatures. Small spontaneous magnetization is observed for $T = 300$ K and B||[2-1-10] (red curve), but vanishes for $T = 200$ K (blue curve). The magnetization values for B||[2-1-10] and B||[0001] at 1 T are almost identical with each other. **b**, Temperature dependence of magnetization for B||[2-1-10] and B||[0001] of 0.1 T.

2. In-plane angle dependence of XMCD spectra

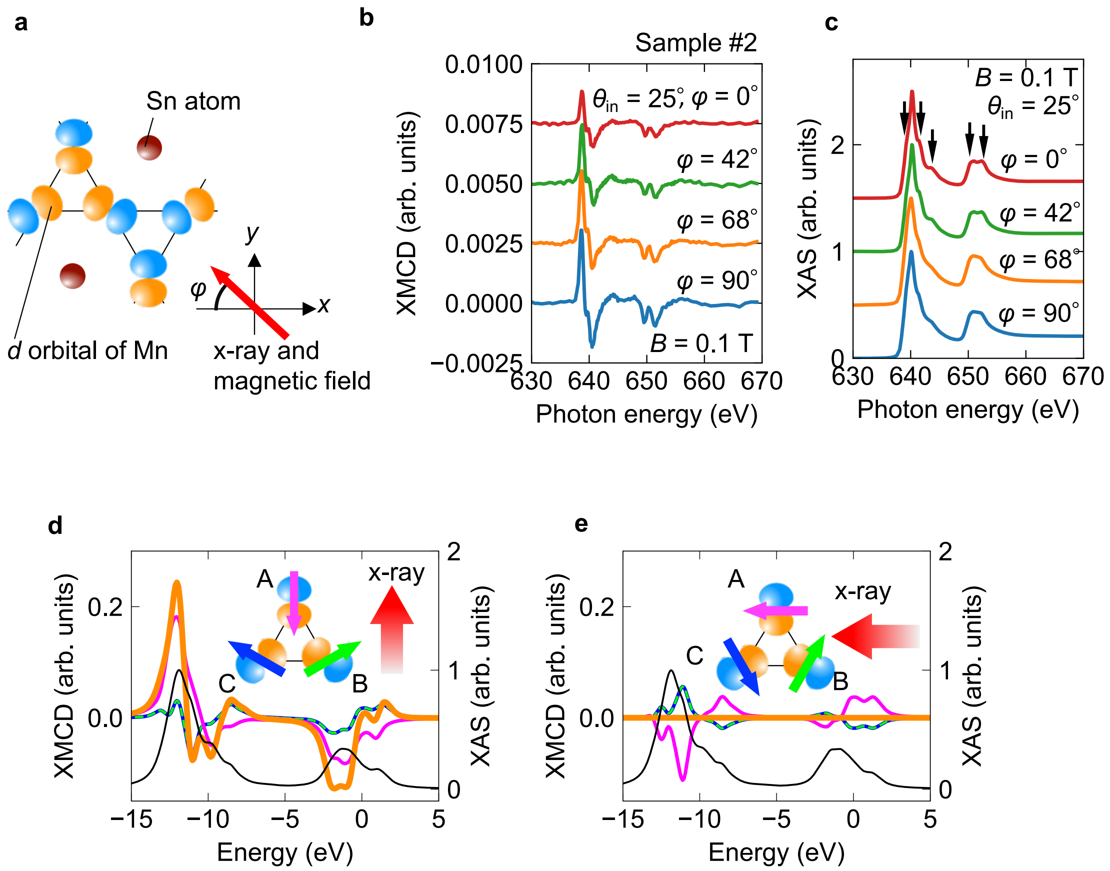

Supplementary Figure S2| In-plane field angle dependence of XMCD spectra and calculated XMCD for various magnetic structures. a, Schematic illustration of orbital arrangement at Mn sites and definition of in-plane angle rotation of x-ray and magnetic field. The directions of external magnetic field and x-ray are parallel to each other within the kagome plane. **b, c**, Experimentally observed in-plane angle (φ) dependence of XMCD spectrum (b) and XAS (c). The XMCD spectral shape is essentially independent of φ, which is consistent with the model calculation (arrows in extended Fig.2d) and the expectation from the group theory [15]. In this experiment, the data for $\varphi = 68°$ was firstly measured, and then φ is changed to 90°, 42°, and 0°. Although a high vacuum of chamber pressure is kept, the slight oxidation of sample surface is hard to avoid due to the intensive x-ray irradiation, which is indeed observed as the growth of multiplet structures correspond to Mn^{2+} in XAS (arrows in extended Fig.2c), i.e., formation of MnO. Since this Mn oxide layer is expected to show no XMCD, the surface oxidation might cause intensity reduction of XMCD signals. **d**, Result of

spectral model calculation for B||[01-10] (≡ y) direction. The x-ray direction is parallel to the magnetic field direction. The shape of total XMCD spectrum (orange line) is same with the case for B||[2-1-10] (≡ x), shown in Fig.4a. **e**, The spectral model calculation for triangle magnetic structure for positive spin chirality. In this case, the XMCD responses from each sublattice are completely compensated, and total XMCD is absent. Note that only the magnetic structure with negative spin chirality is formed in Mn_3Sn, and the magnetic structure with positive spin chirality (extended Fig.2e) is a fictitious structure for comparison.

3. XMCD spectrum calculated by sum rule with first-principles density of states

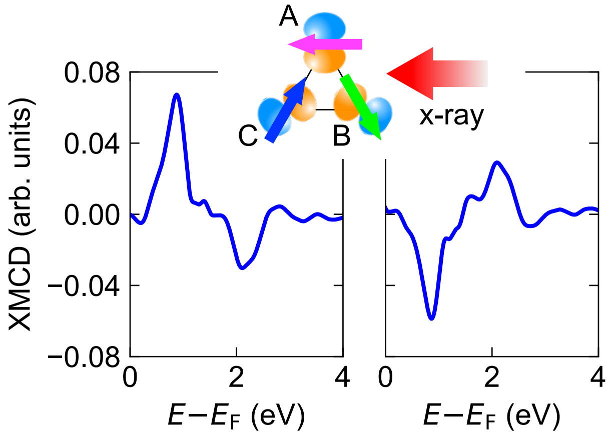

Supplementary Figure S3| XMCD spectra calculated by sum rule with density of states obtained from first-principles calculations. Calculated XMCD spectra near L_3 (left panel) and L_2 (right panel) edges from the relation between XMCD and DOS through the sum rule. DOS obtained from the first-principles calculation was used. The shape of calculated XMCD spectrum for L_3 edge shows good agreement with the experiment.
